# Retraction note: Neuropathological microscopic features of abortions induced by *Bunyavirus*/or *Flavivirus* infections

**DOI:** 10.1186/s13000-016-0568-1

**Published:** 2016-11-02

**Authors:** Javad Javanbakht, Seyed Hossein Mardjanmehr, Abbas Tavasoly, Mohammad Hossein Nazemshirazi

**Affiliations:** 1 Department of Pathology, Faculty of Veterinary Medicine, Tehran University, Tehran, Iran; 2 Department of Molecular Biology, Central Veterinary Laboratory of I.V.O, Tehran, Tehran Iran

## Retraction

The Editor-in-Chief and Publisher have retracted this article [1] because the scientific integrity of the content cannot be guaranteed. An investigation by the Publisher found it to be one of a group of articles we have identified as showing evidence suggestive of attempts to subvert the peer review and publication system to inappropriately obtain or allocate authorship. This article showed evidence of plagiarism (most notably from the articles cited [2–7]) and peer review and authorship manipulation.
